# Extracellular Vesicles from Poor-Outcome Intracerebral Hemorrhage Patients Reveal Limited Reparative Potential in a Preclinical Model

**DOI:** 10.3390/ijms262110648

**Published:** 2025-10-31

**Authors:** Fernando Laso-García, Nerea Díaz-Gamero, Rebeca Gallego-Ruiz, Laura Casado-Fernández, Exuperio Díez-Tejedor, Ángela Calzado-González, Javier Pozo-Novoa, Laura Otero-Ortega, María Alonso de Leciñana, María Gutiérrez-Fernández

**Affiliations:** 1 Neurological Sciences and Cerebrovascular Research Laboratory, Department of Neurology and Stroke Centre, Neurology and Cerebrovascular Disease Group, Neuroscience Area, Hospital La Paz Institute for Health Research—IdiPAZ (La Paz University Hospital-Universidad Autónoma de Madrid), Paseo de la Castellana, 261, 28046 Madrid, Spain; fernilaso.9@gmail.com (F.L.-G.); nereadiazgamero@gmail.com (N.D.-G.); rebegall@ucm.es (R.G.-R.); lauracasf@gmail.com (L.C.-F.); exuperio.diez@salud.madrid.org (E.D.-T.); angelacalzado@gmail.com (Á.C.-G.); javier.pozonovoa@gmail.com (J.P.-N.); oteroortega.l@gmail.com (L.O.-O.); 2 Translational Stroke Laboratory (TREAT), Clinical Neurosciences Research Laboratory (LINC), Health Research Institute of Santiago de Compostela (IDIS), 15706 Santiago de Compostela, Spain

**Keywords:** extracellular vesicles, preclinical study, intracerebral hemorrhage, poor-outcome patients, rat

## Abstract

Extracellular vesicles (EVs) have emerged as potential therapeutic agents for neurological disorders. Their molecular cargo may reflect the clinical status of the donor and has been identified as a biomarker for the cellular damage and repair processes underlying intracerebral hemorrhage (ICH). It has been shown that EVs from patients with favorable outcomes carry a distinct proteomic signature, compared to those from poor outcome patients, which may promote recovery in preclinical models of ICH. We investigated whether intravenously administered EVs isolated from patients with poor outcomes after ICH provide any benefit in a preclinical ICH model. No significant differences were observed in lesion volume between the placebo and treatment groups at 24 h, 72 h, or 28 days post-ICH. Functional assessments using the Rogers and tapered beam walking tests revealed no improvement in motor performance in the treatment group at 24 h, 72 h, 7 d, 14 d and 28 d. Histological analysis at 28 days showed no significant differences in immunofluorescence markers of myelin preservation (MOG, Olig-2), astroglial activation (GFAP), or angiogenesis (VEGF) between groups. In conclusion, EVs derived from patients with poor outcomes after ICH failed to promote functional recovery or modulate markers of injury and repair in a rat model, suggesting few endogenous repair mechanisms.

## 1. Introduction

Intracerebral hemorrhage (ICH) represents the most devastating form of stroke, associated with high rates of mortality and long-term disability [1], with limited therapeutic options. Treatment with mesenchymal stem cells has been shown to reduce neuronal damage and improve outcomes in preclinical models of ICH. These effects appear to result from the enhancement of mechanisms involved in brain plasticity, mediated by the release of trophic factors and other molecules that support reparative processes [2]. These may be caused, at least in part, by the paracrine effect of extracellular vesicles (EVs). It has been proposed that post-ICH recovery reflects the balance between neurotoxic cascades and endogenous repair pathways, and that the molecular cargo of EVs may serve as a biomarker of these processes. EVs are nanosized, membrane-bound structures secreted by every cell, which mediate intercellular communication through the transfer of bioactive molecules, such as proteins or microRNAs. These vesicles play a pivotal role in regulating cellular behavior and have emerged as biomarkers of cellular status and biological processes as well as promising therapeutic agents, particularly in the context of tissue repair. A growing body of evidence has highlighted the therapeutic potential of EVs; in particular, administering EVs from diverse sources has been shown to enhance functional recovery in animal models of stroke, through the promotion of brain plasticity [3,4,5,6].

Along these lines, our research group has previously demonstrated that circulating EVs obtained from patients who experienced favorable spontaneous recovery after ICH significantly improve functional outcomes when administered in an experimental model of ICH. This is probably related to a particular proteomic cargo functionally related to repair processes [7]. This underscores the need for a clearer understanding of the functional implications of EVs from diverse clinical contexts [8,9]. Accordingly, we investigated the effect of EVs from poor-outcome patients in a rat model of ICH, hypothesizing that they would confer no therapeutic effect, unlike EVs from favorable-outcome patients, to strengthen evidence that EV cargo mediates endogenous repair processes and to advance the development of EV content-based therapies.

## 2. Results

Out of the 22 rats initially enrolled in the study (11 in the placebo group and 11 in the active treatment group), 4 (2 from each group) died immediately following ICH induction. Additionally, one animal from the placebo group and another from the treatment group were excluded because they showed no hemorrhage in ultrasound imaging. Following exclusions after ICH induction, each group consisted of 8 animals, and no further attrition occurred during the study. Therefore, each experimental group (placebo and treatment) included *n* = 8 animals. All behavioral assessments (Rogers’s test and tapered beam walking test), imaging evaluations (transcranial B-mode ultrasound), and histological analyses were performed in all animals included in each experimental group (*n* = 8 per group) at the predefined study time-points.

### 2.1. Characterization of Extracellular Vesicles

**Figure 1:**
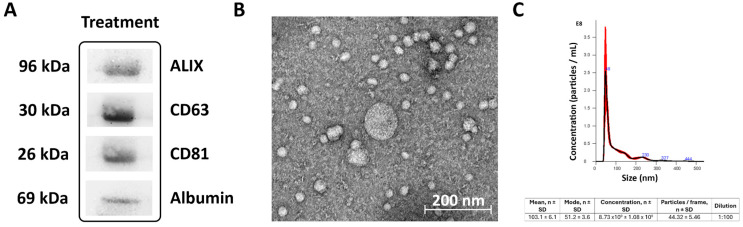
Extracellular vesicle characterization. (**A**) Characterization was performed using Western blotting (WB). (**B**) Transmission electron microscopy (TEM). Scale bar = 200 nm. (**C**) Nanoparticle tracking analysis (NTA).

**Figure 2:**
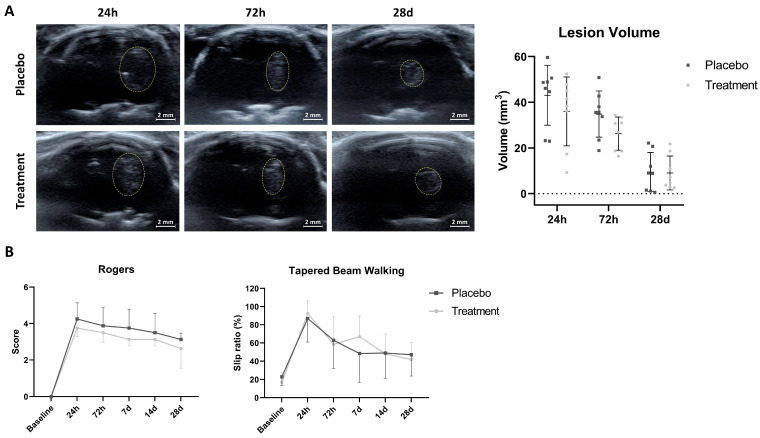
Brain imaging and functional assessment. (**A**) Ultrasound image: representative images of ICH volume and its quantification by B-mode ultrasound at 24 h, 72 h and 28 d post-injury (*n* = 8 rats per group). Scale bar = 2 mm. (**B**) Motor assessment: graphs show performance in the Rogers test and tapered beam walking test at baseline, 24 h, 72 h, 7 d, 14 d and 28 d after ICH (*n* = 8 rats per group). Data are shown as mean ± SD. Statistical analysis was performed using Student’s *t*-test for lesion volume and the Mann–Whitney U test for motor assessment. All *p*-values were corrected using the Benjamini–Hochberg (BH) method to control false discovery rates (FDRs).

**Figure 3:**
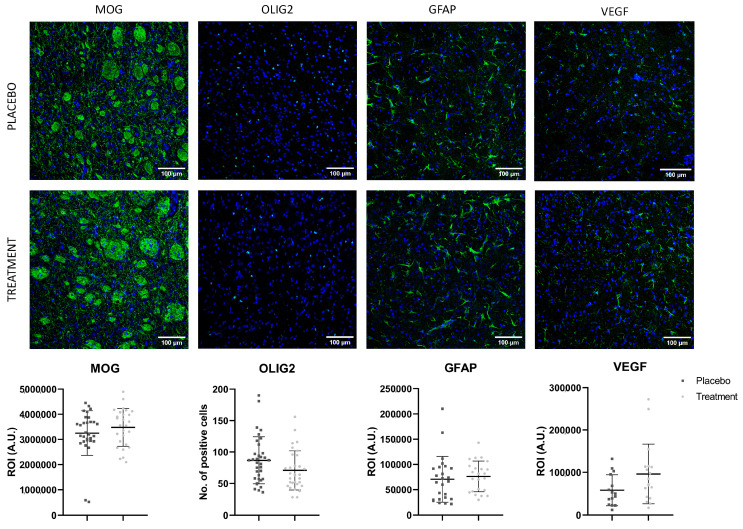
Brain marker expression. Representative images and quantification of MOG, Olig-2, GFAP and VEGF immunofluorescence at 28 d; 3–4 rats per group; images 20×. Scale bar = 100 µm. Data are shown as mean ± SD. Statistical analysis was performed using a linear mixed model (LMM) for continuous response variables (GFAP, VEGF, and MOG) and a generalized linear mixed model (GLMM) for count data (OLIG2). Abbreviations: MOG, myelin oligodendrocyte glycoprotein; Olig-2, oligodendrocyte transcription factor; GFAP, glial fibrillary acid protein; VEGF, vascular endothelial growth factor.

EVs were correctly identified as per the three recommended methods: (1) Western blot analysis of characteristic surface protein biomarkers (Alix, CD63 and CD81) (see Figure 1A); (2) transmission electron microscopy (TEM) to assess morphology and size (<200 nm) (see Figure 1B; (3) nanoparticle tracking analysis (NTA) (see Figure 1C).

### 2.2. No Significant Differences Were Observed in the Lesion Volume Between Groups

Ultrasound-based analysis of ICH volume showed no significant differences between the placebo and treatment groups at baseline, 72 h, or 28 d. Hematoma volume decreased similarly over time in both groups (see Figure 2A and Table 1).

### 2.3. Circulating EVs Derived from Patients with Poor-Outcomes Following ICH Did Not Improve Functional Recovery After Intracerebral Hemorrhage in Rats

The treated group did not show better functional outcome compared to the placebo group in either the Rogers test at baseline, 24 h, 72 h, 7 d, 14 d and 28 d, or the tapered beam walking test at baseline, 24 h, 72 h, 7 d, 14 d and 28 d (see Figure 2B and Table 2).

### 2.4. Lack of Effect of EVs from Patients with Poor Outcome on Histological Markers of Brain Damage and Repair

No significant differences were observed in the immunofluorescence of the histological markers MOG, Olig-2, GFAP and VEGF between the treatment and placebo groups at 28 d (see Figure 3 and Table 3).

## 3. Discussion

This study shows that EVs isolated from the blood of patients with poor outcomes following ICH fail to promote functional recovery and have no observable effect on histological markers for injury and repair in the experimental ICH model. These results are in contrast to earlier investigations with EVs from favorable outcome patients, which enhanced motor function, preserved white matter integrity, and increased histological indicators of reparative processes indicating therapeutic potential [7]. Together, these findings support the hypothesis that favorable outcome EVs promote endogenous repair mechanisms, counterbalancing injury, through the transfer of effector reparative molecules, whereas such mechanisms appear insufficiently potentiated in poor outcome patients, as indicated by the lack of effect of their EVs.

Previous analyses conducted by our group showed that EVs from patients with poor outcomes after ICH contained a greater abundance of proteins linked to inflammatory signaling pathways and pro-apoptotic mechanisms, along with a lower abundance of proteins related to modulation of inflammatory and oxidative stress cascades. The latter were more abundant in patients with spontaneous good outcomes, indicating a proteomic profile associated with protective and brain repair mechanisms [10]. The particular proteomic signature in patients with a poor recovery may reflect the underlying pathological environment and a disbalance between physiological injury and repair mechanisms, thus favoring damage processes. These results are consistent with previous preclinical studies on ICH in rat, in which proteomic analyses of circulating EVs in the post-acute phase reveal different protein signatures; animals exhibiting favorable spontaneous recovery show a greater abundance of proteins mapping to metabolic pathways that mediate protection and brain repair, whereas poor-recovery animals lack this enriched higher abundance, while carrying proteins that reflect persisting neuroinflammation, cellular stress, or impaired repair mechanisms, potentially limiting the EVs’ regenerative potential [7]. In addition, there is a dynamic and phase-specific proteomic signature in circulating EVs following ICH. During the acute phase, EVs are predominantly enriched with proteins associated with cellular stress and damage response, reflecting the immediate aftermath of cerebral injury. As the condition progresses into the post-acute phase, the EV cargo shifts towards proteins involved in debris clearance, tissue remodeling, and repair [11]. These findings emphasize the importance of donor condition and disease state at the time of EV collection when investigating the role of circulating EVs [12]. While EVs are being pursued as a promising field in the development of reparative therapies for neurological disorders, including stroke [13,14,15,16], our results urge caution, as they show not all EVs are equal. Therefore, rigorous characterization of the EV cargo [8,9] and stratification of donor populations based on clinical outcomes should be considered in the development of EV-based treatments.

Although many preclinical studies have reported beneficial effects of EVs in stroke models, increasing experimental evidence indicates that EVs can also exert detrimental effects depending on their cellular origin and molecular cargo. For example, Lombardi et al. showed that microglia-derived EVs under pro-inflammatory conditions impair remyelination by altering astrocytic responses and promoting a pathological milieu [17]. Hirsch et al. discuss how EVs can contribute to microvascular dysfunction and to post-stroke inflammation harnessing neurological recovery [18]. In addition, Ye et al. found that exosomes derived from patients with acute ischemic stroke enriched in miR-27-3p, aggravate brain damage and neuroinflammation [19] and Sprinc et al. demonstrated that certain EVs can increase brain damage if they are enriched with certain mRNAs [20]. Together, these findings highlight that EVs are not universally protective and may contribute to secondary injury under certain pathological contexts. Our results reinforce the concept that the functional impact of EVs is highly dependent on the donor’s clinical status and the molecular composition of the vesicular cargo, which may either support or fail to activate endogenous repair mechanisms.

Additionally, the dose and timing of EV administration are critical variables that may influence therapeutic efficacy. In our study, we selected a single dose of 100 µg of EVs based on previous work from our group, where circulating EVs derived from patients with spontaneous recovery after ICH significantly improved functional outcomes in rats [7]. To maintain consistency and avoid introducing confounding variables, we applied the same dose and single administration when testing circulating EVs from patients with poor outcomes. However, the absence of therapeutic benefit in this context may reflect not only differences in EV cargo but also limitations in the dosing strategy. Recent studies have shown that repeated or fractionated dosing of EVs—particularly those derived from mesenchymal stem cells—can yield superior protective effects compared to single administrations [5,21]. Also, we cannot rule out a possible beneficial effect of earlier administration of treatment. Furthermore, although our isolation protocol followed established guidelines, subtle differences in EV purity or co-isolated components may have influenced efficacy. Future research should explore whether engineering nanoparticles to mimic the EV molecular cargo in different pathophysiological conditions could be therapeutically beneficial in neurological diseases.

Our study has certain methodological limitations that should be acknowledged. First, although it was designed to evaluate the effects of EVs derived from poor-outcome patients using a standardized protocol previously validated in our prior work, including a parallel group of animals treated with favorable-outcome EVs would have enabled a more rigorous assessment of differential therapeutic potential.

Second, extracellular vesicles were isolated using a precipitation method, which, while widely used and suitable for clinical samples, may co-isolate plasma proteins and lipoproteins such as albumin or apolipoproteins. This may reduce EV purity compared to ultracentrifugation or size-exclusion chromatography, potentially influencing the cargo profile and downstream biological effects. Nevertheless, precipitation-based isolation methods are recognized as acceptable in translational studies due to their high yield, reproducibility, and feasibility. Also, we confirmed EV identity through positive markers (Alix, CD63, CD81) using Western blot, and albumin was used as a purity marker. Isolating EVs with high purity remains a complex and challenging task due to their small size and heterogeneity within biological fluids. As ISEV emphasizes, the need for standardized isolation and quality assessment methods is urgent and of the utmost importance to advance fundamental research and biomarker discovery effectively [22].

In conclusion, this study reinforces the hypothesis of a content-specific role of EVs in post-ICH brain repair and suggests EVs from patients with poor outcomes lacking any beneficial effect. These results support EVs molecular content as both a biomarker of donor clinical status and of therapeutic targets in ICH. Further studies are warranted to elucidate the mechanistic characterization of EVs cargo to better understand the molecular determinants of EV-mediated repair after ICH.

## 4. Material and Methods

This preclinical study was conducted at the Neurological Sciences and Cerebrovascular Research Laboratory, Neurology and Cerebrovascular Disease Group, Neuroscience Area of IdiPAZ Health Research Institute, La Paz University Hospital, Madrid, Spain.

### 4.1. Ethics Statement

All experimental protocols were conducted in strict adherence to ethical standards aimed at minimizing animal discomfort and were approved by the Ethics Committee for the Care and Use of Animals in Research (Ref. PROEX 159/17), in accordance with applicable Spanish and European Union legislation (86/609/CEE, 2003/65/CE, 2010/63/EU; Spanish Royal Decrees RD 1201/2005 and RD 53/2013). The animal studies followed established recommendations regarding randomization and statistical power, as outlined by the Stroke Therapy Academic Industry Roundtable (STAIR) [23], RIGOR [24], and HEADS [25] guidelines. In addition, the 3Rs principle (replacement, reduction, and refinement) was followed by including the lowest possible number of animals [26].

The study in humans was approved by the Ethics Committee of La Paz University Hospital. Informed consent was obtained from all participants or their legal representatives prior to inclusion (PI-3093).

### 4.2. Treatment Preparation

The EVs were obtained from blood samples collected 7 days after the onset of symptoms in patients with spontaneous ICH, admitted to the Neurology Department and Stroke Center at Hospital Universitario La Paz in Madrid, Spain, and treated in accordance with current clinical protocols. The EVs were from the patients who showed a poor outcome at 6 months, defined as no significant improvement from NIHSS at baseline and a score of ≥3 on the modified Rankin Scale.

### 4.3. Extracellular Vesicles Isolation and Characterization

Following centrifugation at 3000× *g* for 15 min at 4 °C of serum samples, the EVs were extracted using the ExoQuick Ultra Isolation Kit (System Biosciences, Palo Alto, CA, USA) in accordance with the manufacturer’s protocol, as previously described [7].

EV characterization was performed using three complementary techniques following the recommendations of Minimal Information for Studies of Extracellular Vesicles guidelines [27]: (1) Western blot for protein marker identification; (2) TEM for morphological analysis; (3) NTA for size distribution profiling, as previously described [7].

### 4.4. Preclinical Study

**Figure 4:**
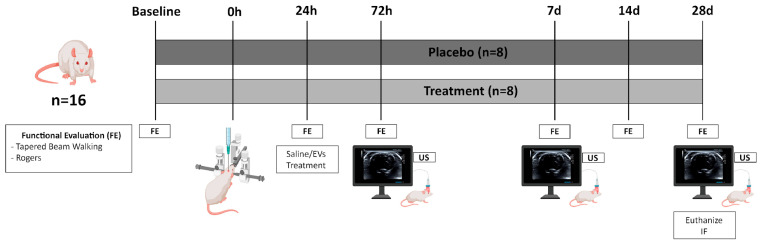
Overview of the preclinical protocol: efficacy study design. Abbreviations: EVs, extracellular vesicles; FE, functional evaluation; IF, immunofluorescence; US, ultrasound.

In this study, Sprague-Dawley rats were used to perform the ICH model via stereotaxic injection of collagenase into the striatum under sevoflurane anesthesia, as previously described [7] (see Figure 4).

To evaluate therapeutic efficacy, male and female rats (ratio of 1:1) were randomly assigned to the following groups: placebo (*n* = 8; ICH plus saline) and treatment (*n* = 8; ICH plus 100 µg of circulating EVs from patients with poor outcomes). The dose of EVs contained approximately 8.93 × 10^10^ particles. The resulting protein-to-particle ratio was approximately 11.2 µg per 10^9^ particles. EVs were administered via tail vein injection at 24 h post-ICH. Group allocation was performed by a researcher blinded to the study outcomes and who generated the randomization sequence, ensuring balanced sex distribution across groups.

Transcranial B-mode ultrasound imaging (Xario 200G, TUS-X200; Canon Medical Systems, Ōtawara, Japan) using a 13 MHz probe was conducted at 72 h, 7 d, and 28 d following ICH induction to assess hemorrhage volume, following the previously validated methodology [28].

Functional assessments using the tapered beam walking and Rogers tests were conducted at baseline and at 24 h, 72 h, 7 d, 14 d, and 28 d post-ICH [7].

Rats were euthanized for histological study at 28 d post stroke by an intracardiac injection of potassium chloride. The brains were fixed by immersion in 4% paraformaldehyde for 24 h and 30% sucrose for 72 h and stored at −80 °C in an optimum cutting temperature compound (Sakura Finetek, Torrance, CA, USA) until the histological experiments were performed.

The peri-hemorrhagic region was examined by immunofluorescence on 10-μm-thick brain sections to evaluate histological markers for tissue injury and repair. An investigator blinded to the study performed the analyses. Myelin preservation was assessed using myelin oligodendrocyte glycoprotein (MOG, 1:50; Millipore, Burlington, MA, USA) and oligodendrocyte transcription factor 2 (Olig-2, 1:450; Millipore, USA) to label oligodendrocyte nuclei. Astrocytic activation in the perilesional area was evaluated using glial fibrillary acidic protein (GFAP, 1:500; Millipore, USA). Angiogenesis was assessed using vascular endothelial growth factor (VEGF, 1:500; Millipore, USA), all followed by Alexa Fluor 488-conjugated secondary antibodies (goat anti-mouse and goat anti-rabbit, 1:750; Invitrogen, Carlsbad, CA, USA). To minimize background normalization bias, all assessments were conducted using identical microscope settings.

All animals included in the two study groups underwent behavioral assessments, and imaging evaluation at the different study time-points and histological analysis after sacrifice. All evaluation procedures (behavioral testing, imaging, and histological analysis) were conducted by investigators blinded to group allocation, that was only revealed after completion of all endpoints.

### 4.5. Statistical Analysis

A power analysis using G*Power 3.1 (Heinrich Heine University Düsseldorf, Düsseldorf, Germany) indicated that, for non-parametric assessment of functional outcomes and lesion volume, a minimum of 8 rats per group was required to achieve a significance level of 5% (alpha) and a statistical power of 80% (1 − beta). Rats that did not develop hemorrhage or that died before the study ended were replaced.

Data are presented as mean standard deviation (SD). Statistical analyses were performed using custom scripts in R version 4.5.0 (R Core Team, Vienna, Austria) and RStudio version 2025.05.0 (Posit PBC, Boston, MA, USA); *p* values < 0.05 were considered statistically significant at a 95% confidence interval. The graphs were obtained using GraphPad Prism 8 (GraphPad software, San Diego, CA, USA).

Normality was assessed using the Shapiro–Wilk test. Variables not meeting the criteria for normality were treated as non-normally distributed.

For the ICH volume and functional evaluation analyses, comparisons between the two groups at different time points were performed using Student’s *t*-test or the Mann–Whitney U test, depending on the normality of the data. All *p*-values were corrected using the Benjamini–Hochberg (BH) method to control false discovery rates (FDRs).

For immunofluorescence analyses, comparisons between the two groups were performed using either a linear mixed model (LMM) for continuous response variables (GFAP, VEGF, and MOG) or a generalized linear mixed model (GLMM) for count data (OLIG2). The normality of residuals was assessed using the Shapiro–Wilk test. When assumptions were not met, the data for continuous variables (LMM) were transformed: logarithmic for GFAP and VEGF, and Box–Cox for MOG.

## Figures and Tables

**Table 1:** Intracerebral hemorrhage volume in the experimental groups.

		Time	Placebo (*n* = 8)	Treatment (*n* = 8)	*p*
ICH volume	US (mm^3^ [mean ± SD])	24 h	43.09 ± 13.14	36.02 ± 15.08	0.502
		72 h	34.86 ± 10.12	26.27 ± 7.27	0.221
		28 d	9.50 ± 8.49	9.05 ± 7.44	0.910

Abbreviations: d, day; h, hour; ICH, intracerebral hemorrhage; SD, standard deviation; US, B-mode ultrasound; SD, standard deviation. Data were compared with Student’s *t*-test or the Mann–Whitney U test based on data distribution normality.

**Table 2:** Functional assessment scores in the experimental groups.

		Time	Placebo (*n* = 8)	Treatment (*n* = 8)	*p*
Functional evaluation	Rogers (score [mean ± SD])	Baseline	0.00 ± 0.00	0.00 ± 0.00	1.000
		24 h	4.25 ± 0.89	3.75 ± 0.46	0.372
		72 h	3.88 ± 0.99	3.50 ± 0.53	0.537
		7 d	3.75 ± 1.04	3.13 ± 0.35	0.372
		14 d	3.50 ± 1.07	3.13 ± 0.35	0.537
		28 d	3.13 ± 0.35	2.63 ± 1.06	0.372
	Tapered Beam Walking (% [mean ± SD])	Baseline	22.91 ± 9.32	16.98 ± 10.42	0.751
		24 h	86.99 ± 25.84	92.52 ± 13.86	0.959
		72 h	62.97 ± 30.58	58.45 ± 30.70	0.959
		7 d	48.45 ± 31.85	66.98 ± 22.86	0.751
		14 d	48.95 ± 27.41	48.03 ± 21.78	0.959
		28 d	47.19 ± 23.33	41.86 ± 18.53	0.959

Abbreviations: d, day; h, hour. Data were compared with Student’s *t*-test or the Mann–Whitney U test based on data distribution normality.

**Table 3:** Histological markers of brain damage and repair in the experimental groups.

		Placebo (*n* = 3–4)	Treatment (*n* = 3–4)	*p*
Myelin marker	MOG (ROI (A.U.) [mean ± SD])	3,252,835.10 ± 886,143.92	3,479,484.30 ± 746,306.94	0.521
Oligodendrocyte marker	OLIG-2 (No. of positive cells [mean ± SD])	86.79 ± 37.38	70.87 ± 31.08	0.054
Astrocyte marker	GFAP (ROI (A.U.) [mean ± SD])	70,285.12 ± 45,506.14	76,014.09 ± 30,480.15	0.646
Vascular marker	VEGF (ROI (A.U.) [mean ± SD])	57,867.34 ± 36,448.18	96,183.92 ± 70,429.66	0.156

Abbreviations: GFAP, Glial Fibrillary Acidic Protein; MOG, myelin oligodendrocyte glycoprotein; ROI (A.U.), region of interest measured in arbitrary units; arbitrary units; VEGF, vascular endothelial growth factor. Data were analyzed using either a Linear Mixed Model (LMM) for continuous response variables (GFAP, VEGF, and MOG) or a Generalized Linear Mixed Model (GLMM) for count data (OLIG2). The normality of residuals was assessed using the Shapiro–Wilk test. When assumptions were not met, data for continuous variables (LMM) were transformed: logarithmic for GFAP and VEGF, and Box–Cox for MOG.

## Data Availability

The original contributions presented in this study are included in the article. Further inquiries can be directed to the corresponding author(s).

## Abbreviations

The following abbreviations are used in this manuscript:

ICH	Intracerebral hemorrhage
EVs	Extracellular vesicles
TEM	Transmission electron microscopy
NTA	Nanoparticle tracking analysis
SD	Standard deviation
BH	Benjamini–Hochberg
FDR	False discovery rates
LMM	Linear mixed model
